# Improved Clinical and Radiological Outcomes with Double-Cage Biportal Endoscopic Transforaminal Lumbar Interbody Fusion: A Comparative CT-Based Study

**DOI:** 10.3390/diagnostics15202652

**Published:** 2025-10-21

**Authors:** Yu-Hao Huang, Jwo-Luen Pao

**Affiliations:** Department of Orthopedic Surgery, Far Eastern Memorial Hospital, New Taipei City 22060, Taiwan; oshyh1005@gmail.com

**Keywords:** minimally invasive surgery, biportal endoscopy, lumbar interbody fusion, fusion cage, computed tomography, treatment outcomes

## Abstract

**Background/Objectives**: When transitioning from an older surgical technique to a newer one, we expect improved treatment outcomes and fewer complications. However, direct comparative studies to confirm these advantages are often lacking. Tubular minimally invasive transforaminal lumbar interbody fusion (MISTLIF) has been widely used, but limitations in visualization and endplate preparation may compromise fusion quality. Biportal endoscopic TLIF (BETLIF), a more recent alternative, offers enhanced magnification and superior hemostasis. Still, CT-based comparative data on fusion integrity remain limited. To evaluate the clinical and radiological outcomes following a chronological transition from MISTLIF to BETLIF, using thin-slice CT to assess fusion integrity. **Methods**: This retrospective study analyzed 179 patients treated by a single surgeon between January 2018 and May 2021. The first 90 cases underwent MISTLIF, followed by 89 BETLIF procedures. Clinical outcomes included Visual Analog Scale (VAS), Oswestry Disability Index (ODI), and Japanese Orthopedic Association (JOA) scores. Radiological assessments at one year postoperatively (X-ray and thin-slice CT) included disc height, segmental lordosis, Bridwell fusion grade, cage subsidence, and subchondral osteolysis. **Results**: BETLIF was associated with significantly shorter hospital stays (5.7 vs. 7.4 days) and fewer transfusions (0% vs. 14.7%). BETLIF showed significantly better ODI (12.7 vs. 23.5), JOA scores (26.4 vs. 20.6), and comparable VAS improvement. Radiologically, BETLIF had significantly higher fusion rates (93.3% vs. 82.4%), greater disc height restoration, and lower rates of cage subsidence (5.0% vs. 13.7%) and osteolysis (13.3% vs. 52.9%). **Conclusions**: BETLIF demonstrated superior clinical and radiological outcomes, likely due to enhanced endoscopic visualization and precise endplate preparation.

## 1. Introduction

Lumbar interbody fusion is a well-established surgical procedure for patients with degenerative lumbar conditions. Among various posterior approaches, transforaminal lumbar interbody fusion (TLIF) is widely utilized because of its unilateral access to the disc space and preservation of posterior elements.

Minimally invasive TLIF (MISTLIF), introduced by Foley and Lefkowitz in 2002, utilizes a tubular retractor system to access the surgical field while minimizing soft tissue trauma [1,2]. This technique provides well-known benefits, including reduced blood loss, reduced postoperative pain, shorter hospital stay, and comparable clinical outcomes compared to open TLIF. However, technical challenges, such as a narrow surgical corridor and limited visualization, can affect disc preparation and graft placement, potentially compromising fusion results [1,3,4,5].

Unilateral biportal endoscopic (UBE) spinal surgery has emerged as a promising alternative that offers enhanced visualization, excellent bleeding control, and ergonomic handling of surgical instruments [6,7,8,9]. UBE-based transforaminal lumbar interbody fusion, also known as biportal endoscopic TLIF (BETLIF), has demonstrated promising results in both clinical and radiological domains [10,11,12]. BETLIF allows for radical disc space preparation under direct endoscopic visualization, preservation of the subchondral bony endplate, generous graft placement, and the insertion of large or double interbody cages, all of which may contribute to improved fusion integrity and lower risk of cage subsidence [10,13,14].

Despite these theoretical advantages, comparative studies assessing the fusion quality and clinical efficacy of BETLIF versus MISTLIF remain scarce. The existing literature reports that the treatment outcomes of BETLIF are either compatible or inferior to those of MISTLIF [11,15,16,17]. Implementing new techniques requires investment in equipment and training and involves a learning curve that may initially increase complication rates [18,19,20]. Therefore, it is crucial to determine whether BETLIF yields superior outcomes that justify its broader clinical adoption.

Although plain radiographs are often used to assess fusion, thin-slice computed tomography (CT) with multiplanar reconstruction is currently the most reliable method for evaluating the interbody fusion status [21,22,23]. However, this modality is underutilized in assessing the treatment outcomes of BETLIF. Additionally, key radiographic parameters, such as cage subsidence and subchondral osteolysis, which are potential markers of pseudoarthrosis, are frequently overlooked or misinterpreted as part of the normal fusion process [24,25,26].

This study aims to compare the clinical and radiographic outcomes between MISTLIF and BETLIF using standardized CT-based fusion criteria. We hypothesized that BETLIF results in superior fusion quality and clinical outcomes compared with MISTLIF.

## 2. Materials and Methods

### 2.1. Patient Selection

After receiving approval from the Institutional Review Board, this retrospective study included all patients who underwent minimally invasive lumbar interbody fusion performed by the corresponding author at our institution from January 2018 to May 2021. Surgical indications comprised lumbar disc degeneration with radiographic evidence of segmental instability, presenting as mechanical lower back pain, with or without neurological symptoms or radiculopathy.

Segmental instability was defined as one or more of the following criteria: (1) spondylolisthesis exceeding Meyerding grade II; (2) anterior–posterior translation > 4 mm on dynamic lateral radiographs; (3) segmental disc angle change > 10° on flexion-extension views; or (4) presence of a vacuum disc on plain radiographs with corresponding facet effusion on MRI.

Exclusion criteria included: (1) prior lumbar spinal surgery, (2) spinal infection or neoplastic disease, and (3) follow-up duration < 12 months.

A total of 179 patients (45 males, 134 females) were included in this study. The first 90 patients underwent MISTLIF using a tubular retractor system, while the following 89 patients underwent BETLIF via the UBE approach.

### 2.2. Surgical Techniques

**Figure 1:**
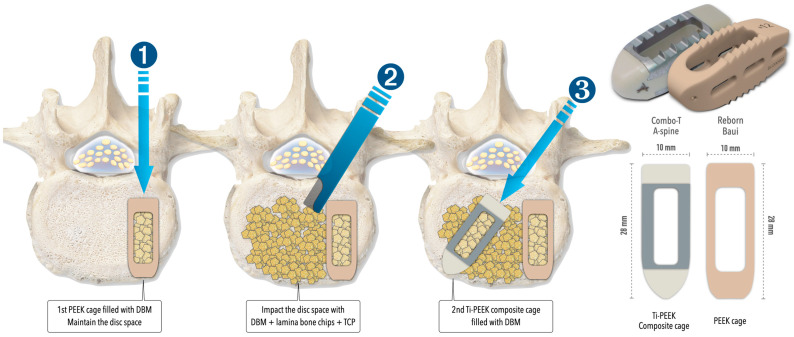
The illustrations explain the insertion of double cages and the specifications of the cages used in both MISTLIF and BETLIF. DBM = demineralized bone matrix; PEEK = polyetheretherketone; Ti = Titanium; TCP = tricalcium phosphate.

The two procedures share similar elements in terms of anesthesia, patient positioning, sterilization, neural decompression, instrumentation, and fixation. Both used a double-cage construct, featuring one PEEK cage (Reborn^®^, Baui, Taipei, Taiwan) and one Ti-PEEK composite cage (Combo-T^®^, A-Spine, Taipei, Taiwan). The bone graft material includes 3 mL of demineralized bone matrix (SurFuse^®^, HansBiomed, Daejeon, Republic of Korea), autografts from laminotomy/facetectomy, and β-tricalcium phosphate blocks (Figure 1). Fixation is performed under fluoroscopic guidance with cannulated pedicle screws (Smartloc, A-Spine, Taipei, Taiwan), which have breakable long barrels designed to facilitate rod insertion and spondylolisthesis reduction using the cantilever technique. The key distinctions between these two approaches are summarized in Table 1 and explained below:

#### 2.2.1. Bleeding Control and Irrigation

BETLIF is performed under continuous saline irrigation to maintain the hydrostatic pressure, facilitate debris clearance, and suppress bleeding. Hemostasis is achieved using radiofrequency wands (ArthroCare^®^, Austin, TX, USA) and bone wax. Therefore, a waterproof draping and drainage system is essential. In contrast, MISTLIF is performed in air, with hemostasis relying on suction and electrocautery, which often leads to higher intraoperative blood loss.

#### 2.2.2. Working Channel and Soft Tissue Management

**Figure 2:**
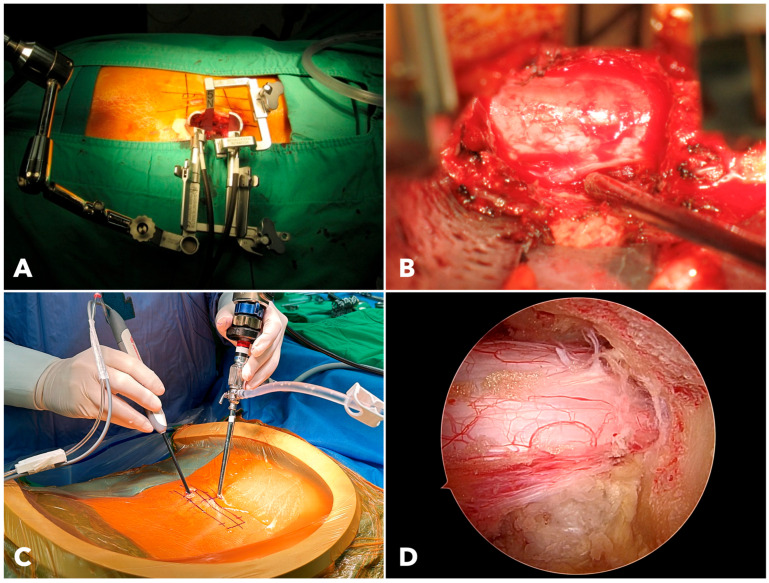
(**A**) The setup of the extendable tubular retractor system used for MISTLIF. (**B**) The surgical field of MISTLIF viewed under the surgical microscope. (**C**) The operating scene of BETLIF demonstrates the handling of endoscopes and surgical instruments. (**D**) The clear and magnified surgical field of BETLIF viewed under the endoscope.

MISTLIF utilizes a 26 mm diameter tubular retractor system (METRx X-TUBE^®^, Medtronic Sofamor Danek, Minneapolis, MN, USA). Soft tissues inside the tube must be removed. In contrast, BETLIF does not require a fixed retractor. The hydrostatic pressure creates a dynamic working space after muscle detachment and coagulation, minimizing tissue trauma (Figure 2).

#### 2.2.3. Decompression and Visualization

In MISTLIF, visualization is limited by the tubular corridor, which requires table tilting for contralateral access. In contrast, BETLIF allows the surgeon to advance a 4 mm endoscope into the spinal canal. The 30° lens further expands the field of view without ergonomic compromise. The endoscopic system and saline medium provide a clear, magnified image, improving decompression efficiency.

#### 2.2.4. Disc Space Preparation

**Figure 3:**
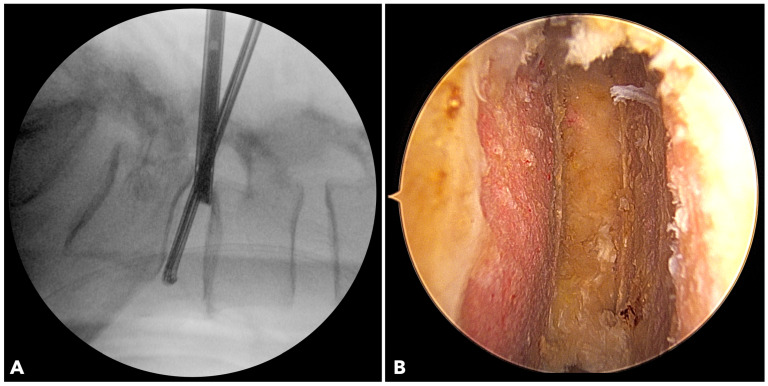
(**A**) The fluoroscope image shows the endoscope being inserted into the disc to evaluate the final results of disc space preparation. (**B**) The endoscopic photo verifies a perfect disc space preparation with no damage to the bony endplate.

In BETLIF, direct endoscopic visualization enables radical discectomy while preserving the bony endplate. Sharp disc shavers and curettes are avoided in favor of blunt disc spreaders and specially designed endplate strippers. The endoscope is inserted into the disc space to ensure complete removal of the cartilaginous endplate (Figure 3). In MISTLIF, disc space preparation relies on tactile feedback via serial disc shavers and curettes, increasing the possibility of inadvertent endplate violation.

### 2.3. Clinical and Radiological Outcome Assessment

Clinical and demographic data were retrieved from the medical records, including age, sex, body mass index, comorbidities, operative time, intraoperative blood loss, transfusion, hospital stay, and complications. Patient-reported outcomes were evaluated using the Visual Analog Scale (VAS) for back and leg pain, the modified Oswestry Disability Index (ODI), and the Japanese Orthopedic Association (JOA) score. Assessments were performed preoperatively and at 3, 6, and 12 months postoperatively, with annual follow-up.

In addition to analyzing mean score differences, we evaluated the proportion of patients achieving the minimum clinically important difference (MCID) for each functional outcome measure. The following MCID thresholds, based on established literature, were applied: 2.1 for VAS for back pain, 2.8 for VAS for leg pain, 14.9 for ODI, and 2.0 for JOA score [27,28].

Radiographic assessments included anteroposterior and lateral lumbar radiographs taken before surgery and at 1, 3, 6, and 12 months postoperatively. Flexion-extension lateral views were obtained preoperatively, at 12 months, and then every one to two years afterward to evaluate fusion status, segmental stability, and adjacent segment degeneration. Disc height was defined as the mean of anterior and posterior disc space measurements. Segmental lordosis refers to the angle between the superior endplates of the upper and lower vertebrae of the fusion segment [29]. All patients underwent magnetic resonance imaging (MRI) of the lumbar spine before the surgery.

**Figure 4:**
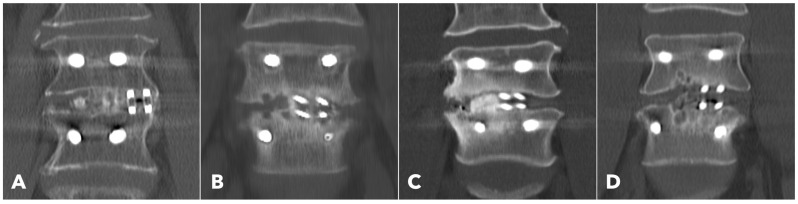
Postoperative 1-year CT reconstruction to evaluate the Bridwell fusion grade. (**A**) Grade I solid fusion with remodeling of the trabeculae. (**B**) Grade II fusion with bridging bone formation and no radiolucency between the cages and the endplates. (**C**) Grade III fusion with radiolucency between the bone graft and endplate. (**D**) Grade IV fusion with resorption of the bone graft.

**Figure 5:**
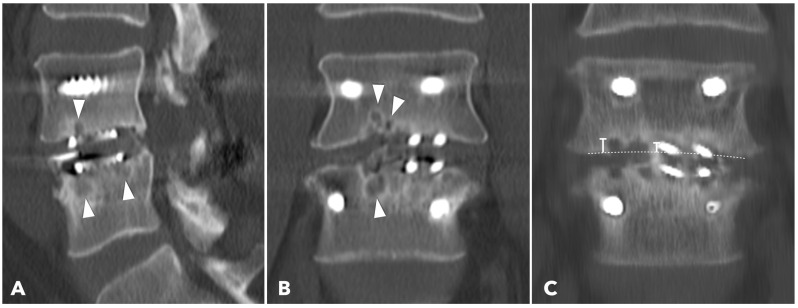
(**A**,**B**) 1-year CT reconstruction revealed the presence of subchondral osteolysis or endplate cyst formation (white arrowheads). (**C**) The measurement of cage subsidence on the coronal CT reconstruction image.

Lumbar spine computed tomography (CT) with 3 mm thin-slice coronal and sagittal reconstructions was performed one year after the surgery. Fusion status was assessed using the Bridwell grading system based on bone window images (Figure 4). Grades I and II were defined as successful fusion. Cage subsidence was defined as sinking > 2 mm beyond the bony endplate [24,30]. The presence of subchondral osteolysis was also recorded as a potential indicator of pseudoarthrosis (Figure 5).

### 2.4. Statistical Analysis

Continuous variables are expressed as mean ± standard deviation. Between-group comparisons were performed using Student’s *t*-test for continuous data and the chi-square test for categorical data. The null hypothesis is that BETLIF does not yield superior outcomes compared to MISTLIF. To address the issue of multiple hypothesis testing, *p*-values were adjusted using the Benjamini–Hochberg procedure to control the false discovery rate (FDR). Both raw and FDR-adjusted *p*-values (*q*-values) were reported to enhance interpretability and maintain statistical rigor. Adjusted *p*-values (*q*-values) < 0.05 were considered statistically significant.

## 3. Results

There were 90 patients in the MISTLIF group and 89 in the BETLIF group who fulfilled the selection criteria, with 114 fused segments per group. There were no statistically significant differences between groups in demographic characteristics, follow-up duration, preoperative diagnoses, or surgical levels (Table 2).

The rate of intraoperative or postoperative blood transfusion was significantly higher in the MISTLIF group than in the BETLIF group (14.7% vs. 0%, *q* < 0.001). The mean hospital stay was also significantly longer for MISTLIF patients (7.4 ± 2.3 days) than for BETLIF patients (5.7 ± 1.1 days, *q* < 0.001) (Table 3).

Preoperative clinical parameters, including back and leg VAS scores, ODI, and JOA scores, were comparable between the groups (all *q* > 0.05), indicating similar baseline functional status. Both groups experienced significant postoperative improvement in back and leg pain, with no statistically significant intergroup differences at the final follow-up (VAS back pain, *q* = 0.850; VAS leg pain, *q* = 0.653). However, the BETLIF group demonstrated superior postoperative functional recovery, as evidenced by a significantly lower ODI score (12.7 ± 16.1 vs. 23.5 ± 14.4, *q* < 0.001) and higher JOA score (26.4 ± 3.2 vs. 20.6 ± 2.5, *q* < 0.001) (Table 3 and Table 4).

The proportion of patients achieving MCID was generally higher in the BETLIF group. Specifically, MCID attainment rates for ODI (83.1% vs. 73.3%) and JOA score (97.7% vs. 88.9%) were notably higher compared to MISTLIF. After applying the Benjamini–Hochberg correction, MCID attainment for JOA score remained statistically significant (*q* = 0.042), while the differences in VAS (back), VAS (leg), and ODI did not reach statistical significance (*q*-values = 0.140, 0.272, and 0.179, respectively). This suggests that while both groups showed functional improvement, BETLIF achieved clinically meaningful recovery more consistently, especially in neurological function, as reflected by the JOA score.

The overall complication rate was low and comparable between groups. Dural tears, epidural hematomas, and transient neurological deficits occurred at similar frequencies without a significant difference. Pedicle screw malposition was observed only in the BETLIF group (2.25%, *q* = 0.230), whereas screw loosening occurred only in the MISTLIF group (3.33%, *q* = 0.149). The reoperation rates were also low and similar between groups (1.1% vs. 2.25%, *q* = 0.673). Three patients required a re-operation. One patient in the BETLIF group required revision for screw malposition-induced motor weakness. One patient in each group required a reoperation for postoperative epidural hematoma and motor weakness. All the affected patients achieved near-complete recovery following revision surgery (Table 3).

Radiographic analysis revealed comparable preoperative disc heights between the groups (5.9 ± 1.0 mm vs. 6.1 ± 1.7 mm, *q* = 0.445). Both groups exhibited significant postoperative increases in disc height; however, the BETLIF group achieved a significantly greater postoperative disc height (10.5 ± 0.9 mm vs. 9.6 ± 1.0 mm, *p* < 0.001) and greater disc height restoration (4.4 ± 1.5 mm vs. 3.7 ± 1.5 mm, *q* = 0.005).

Segmental lordosis improved significantly in both groups postoperatively, with no statistically significant differences between groups in the magnitude of change (6.2 ± 3.7° vs. 6.1 ± 2.0°, *q* = 0.937) or in the pre- and postoperative angles (*q* = 0.339 and 0.173, respectively).

**Figure 6:**
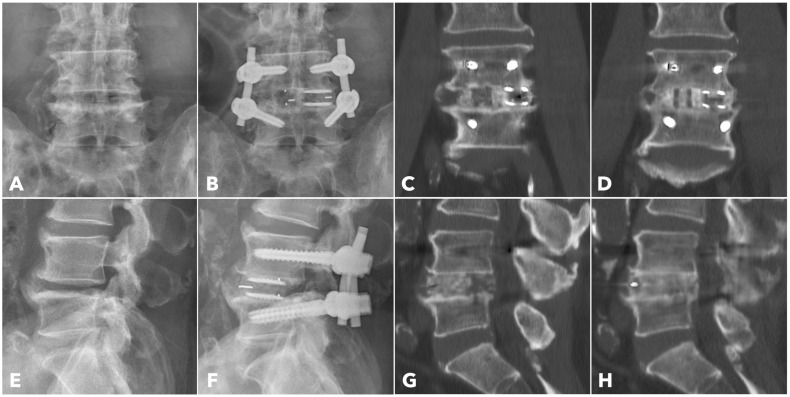
A 67-year-old male patient underwent BETLIF at L4-5 due to recurrent lumbar disc herniation and painful disc degeneration (**A**,**E**). The postoperative X-rays showed restoration of the disc height with double cages in the disc space (**B**,**F**). The immediate postoperative CT scan demonstrated the cage positions and no violation of the bony endplates (**C**,**G**). The 1-year CT scan revealed Bridwell grade I solid fusion with remodeling of the bone graft (**D**,**H**).

**Figure 7:**
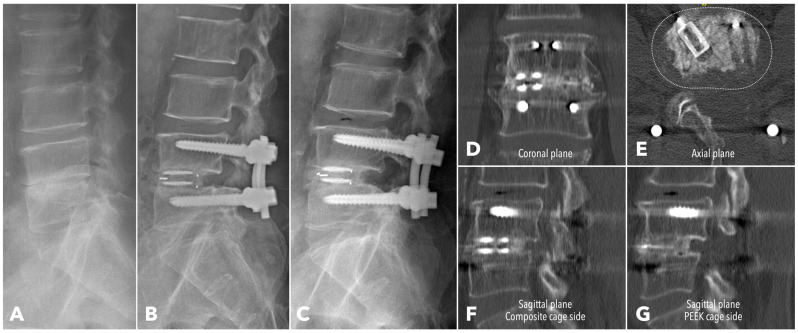
A 62-year-old male patient underwent BETLIF at L4-5 due to painful disc degeneration with restoration of the disc height after the surgery (**A**,**B**). The postoperative 6-month X-ray showed consolidation of the bone graft (**C**). The 1-year CT scan revealed Bridwell grade I solid fusion with remodeling of the bone graft at both coronal plane (**D**) and sagittal plane (**F**,**G**) reconstructions. The axial plane reconstruction (**E**) demonstrated the cage footprint and the fusion bed. The white dashed line indicates the margin of the vertebral body.

Among the segments evaluated by CT at one year (56.7% in MISTLIF vs. 67.4% in BETLIF, *q* = 0.216), the BETLIF group showed significantly higher rates of bridging bone in the sagittal (96.6% vs. 88.2%, *p* = 0.027) and coronal planes (93.3% vs. 82.4%, *p* = 0.025). The overall fusion success rate (Bridwell Grade I or II) was significantly higher in the BETLIF group (93.3% vs. 82.4%, *p* = 0.025). However, these statistical significances were eliminated after applying the Benjamini–Hochberg correction to control the FDR. Furthermore, a substantially higher proportion of BETLIF cases were classified as Bridwell Grade I (73.3% vs. 19.6%, *q* < 0.001), indicating superior fusion quality (Figure 6 and Figure 7).

Subchondral osteolysis was significantly more frequent in the MISTLIF group (52.9% vs. 13.3%, *q* < 0.001). Cage subsidence was also significantly more prevalent in the MISTLIF group (13.7% vs. 5.0%, *p* = 0.023), although most patients in both groups exhibited no evidence of subsidence (Table 5). Again, the statistical significance was no longer significant after applying the Benjamini–Hochberg correction to control the FDR.

## 4. Discussion

This study provides a comprehensive comparison of biportal endoscopic TLIF (BETLIF) and minimally invasive tubular TLIF (MISTLIF) in terms of clinical and radiological outcomes, with a particular emphasis on fusion quality assessed using thin-slice CT scans, which is currently considered the gold standard for evaluating interbody fusion [21,23]. Our findings suggest that BETLIF yields superior results to MISTLIF in several critical aspects, including blood loss, hospitalization duration, functional recovery, and radiographic evidence of fusion.

BETLIF was associated with significantly lower rates of intraoperative or postoperative blood transfusion, with none of the BETLIF patients requiring transfusion compared with 14.7% in the MISTLIF group. Continuous saline irrigation intrinsic to the UBE technique enables superior hemostasis by maintaining hydrostatic pressure and suppressing venous oozing. This facilitates a clear visual field for safe tissue dissection and reduces transfusion-related risks such as immunologic reactions or infections [31].

Hospitalization time was also significantly reduced in the BETLIF group. Although both approaches utilize the Wiltse intermuscular corridor to minimize tissue disruption, BETLIF eliminates the need for a tubular retractor and relies on dynamic saline irrigation to maintain working space. This allows for smaller incisions, less muscle dissection, and potentially faster postoperative recovery [3,10,16,17].

Pain relief and improved functional status were observed in both groups after surgery. However, BETLIF demonstrated significantly better results at the final follow-up, indicated by lower ODI and higher JOA scores. These improvements are likely due to the minimal soft tissue injury from the endoscopic approach and minimal bleeding during surgery [9,10,12,32]. Unlike the significant improvements in ODI and JOA scores, there was no notable difference in VAS scores for leg and back pain between the groups at final follow-up. ODI and JOA scores assess broader functional disability and are likely more sensitive to the benefits of BETLIF. In contrast, VAS scores for back and leg pain are more influenced by segmental stabilization and effective neural decompression, which was effective in both techniques.

Our analysis of MCID attainment highlights the clinical relevance of the observed outcome differences. Although the mean ODI and JOA scores significantly improved in the BETLIF group, only the JOA MCID attainment rate remained statistically significant after FDR correction. This finding supports the notion that BETLIF may provide a more robust improvement in neurological recovery, which may be attributed to better endoscopic visualization and more precise decompression techniques. The application of the Benjamini–Hochberg method further strengthens the validity of our results by mitigating the risk of false-positive findings from multiple comparisons.

Radiographic analysis further favored BETLIF. CT-based evaluation demonstrated significantly higher fusion rates in the BETLIF group (93.3% vs. 82.4%), with a markedly greater proportion achieving Bridwell Grade I fusion (73.3% vs. 19.6%). This improvement is especially notable, given that all other surgical parameters (i.e., cage material, bone graft composition, and fixation technique) were identical between the groups. Thus, the superior outcomes of BETLIF can be reasonably attributed to enhanced disc space preparation and preservation of the bony endplate under direct endoscopic visualization.

In MISTLIF, disc preparation is performed in air, with visualization often hindered by bleeding and constrained by the retractor’s corridor. Surgeons must rely on tactile feedback, which may unintentionally damage the bony endplate and increase the risk of cage subsidence or pseudoarthrosis. Some studies have attempted to reduce endplate damage by using fluoroscopy. However, when damage was visible on the fluoroscope, it had already occurred. In a cadaveric study, Tatsuma et al. demonstrated that 48% of the endplate area was damaged after MISTLIF [33]. In contrast, BETLIF allows clear visualization of the entire disc space under saline medium, enabling precise cartilage removal without damage to the subchondral bone, a critical factor for promoting fusion integrity [34,35].

Cage subsidence was significantly less frequent in BETLIF (5.0%) than in MISTLIF (13.7%, *p* = 0.023), although such significance was mitigated after applying the Benjamini–Hochberg correction. While some authors argue that mild subsidence may represent a physiological adaptation during fusion, excessive subsidence can lead to loss of disc height and segmental lordosis, recurrent stenosis, and complications related to hardware [24,30,36,37]. Increasing the cage footprint using double cages is a reasonable approach to enhance initial segmental stability, improve load distribution, and ensure graft-to-endplate contact [10,36,38,39,40,41]. In posterior approaches, neural elements often limit the size of the cage. However, the UBE technique facilitates safe and effective neural decompression, including the release of epidural adhesions, thereby expanding the accessible corridor. The use of “sentinel pins” to shield neural structures allows for the insertion of larger cages even in severely collapsed disc spaces [10,42]. The BETLIF’s emphasis on endplate preservation, combined with the ability to insert larger cages via safe neural retraction and annular release, may explain the lower subsidence rate and improved disc height restoration in this group.

Although both groups showed significant postoperative increases in segmental lordosis, no intergroup differences were observed. This finding is consistent with previous studies that have demonstrated posterior fusion techniques to be less effective in restoring global sagittal alignment than anterior approaches [29,43]. In our cohort, spinal fusion was primarily indicated for instability and neural compression, rather than correction of deformity. Techniques such as anterior cage positioning and cantilever reduction may account for the segmental lordosis gains observed; however, assessing their impact on global alignment requires a prospective long-term follow-up study, which is beyond the scope of this study [38,44,45,46].

This study has several limitations. First, as a retrospective study, it was subject to inherent selection and follow-up biases, particularly in the subgroup of patients (56.7% and 67.4% in each group) who underwent CT evaluation. Second, the sample size may be insufficient to detect rare or long-term complications. Third, the follow-up period might not fully reflect the durability of outcomes or long-term alignment effects. BETLIF, by minimizing muscle detachment and preserving posterior ligamentous structures, may reduce abnormal biomechanical stress at adjacent levels. Long-term follow-up is necessary to verify its protective effect against adjacent segment degeneration. Nevertheless, standardized surgical techniques, consistent instrumentation and fusion materials, and uniform postoperative protocols minimize confounding factors, enabling a focused comparison of the two approaches.

## 5. Conclusions

Both MISTLIF and BETLIF are safe and effective minimally invasive techniques for lumbar interbody fusion that yield favorable clinical and radiological outcomes. However, this study demonstrates that BETLIF provides significantly improved functional recovery, higher fusion rates, and better fusion quality than MISTLIF. These advantages are primarily attributable to the inherent strengths of the biportal endoscopic approach, including enhanced visualization, superior hemostasis, and the ability to perform meticulous disc space preparation under direct endoscopic guidance. Given these findings, BETLIF represents a promising advancement in minimally invasive spine surgery and may serve as a preferred technique for achieving reliable and high-quality lumbar fusion.

## Figures and Tables

**Table 1:** Summary of MISTLIF and BETLIF.

	MISTLIF	BETLIF
**Approach**	Wiltse’s mini-open approach	Wiltse’s mini-open approach
**Skin incision**	Lateral pedicle line	1–2 cm lateral to lateral pedicle line
**Surgical wounds**	Two	Three, including one 5 mm surgical wound as the endoscopic portal
**Wound size**	3–4 cm for 1-segment fusion 5–6 cm for 2-segment fusion	2–2.5 cm for 1-segment fusion 3–4 cm for 2-segment fusion
**Retractor**	Expandable tubular retractor system, 26 mm diameter, 4–8 cm length	No retractor
**Medium**	Air	Normal saline
**Equipment**		
Visualization	Loupe or surgical microscopic system	Endoscopic system of 720 p, 1080 p, or 4K resolution, 0 or 30-degree endoscope
Light source	Fiber optic light cable attached to the retractor	Light cable from the endoscopic system
Hemostasis	Unipolar and bipolar electrocautery	Radiofrequency wand
Bone removal	High speed drill system	High speed drill system
Disc preparation	Serial disc shavers, curettes, and pituitary rongeurs	Blunt disc spreaders, endplate strippers, pituitary rongeurs
Operation table	Radiolucent spine table	Radiolucent spine table
Localization	Fluoroscopy	Fluoroscopy
**Implants**		
Fusion cages	Two, one PEEK and one composite Ti-PEEK cage	Two, one PEEK and one composite Ti-PEEK cage
Bone grafts	3 mL of DBM putty and autograft from facetectomy and laminotomy, β-TCP blocks	3 mL of DBM putty and autograft from facetectomy and laminotomy, β-TCP blocks
Fixation	Fluoroscopy guide Transpedicle screws with reduction extension barrel, bilateral fixation	Fluoroscopy guide Transpedicle screws with reduction extension barrel, bilateral fixation

The major differences between groups are highlighted with a light grey background color. MISTLIF = minimally invasive transforaminal lumbar interbody fusion; BETLIF = biportal endoscopic transforaminal lumbar interbody fusion; DBM = demineralized bone matrix; PEEK = polyetheretherketone; Ti = Titanium; β-TCP = beta-tricalcium phosphate.

**Table 2:** Demographic characteristics between groups.

	MISTLIF	BETLIF	*p*-Value	*q*-Value *
**Patient number**	90	89		
**Age (year)**	66.9 ± 9.6	64.7 ± 8.7	0.110 ^†^	0.181
**Gender**				
Male	28 (31.1%)	17 (19.1%)	0.093 ^‡^	0.164
Female	62 (68.9%)	72 (80.9%)		
**Diagnosis**				
Degenerative spondylolisthesis	77 (85.6%)	83 (92.2%)	0.238 ^‡^	0.337
Degenerative disc disease	10 (11.1%)	4 (4.4%)		
Degenerative scoliosis	3 (3.3%)	4 (4.4%)		
**Fusion segments**	114	114		
1-segment	69 (60.5%)	66 (57.9%)	0.780 ^‡^	0.865
2-segment	18 (15.8%)	21 (18.4%)		
3-segment	3 (2.6%)	2 (1.8%)		
**Follow-up (months)**	18.8 ± 6.4	17.9 ± 5.8	0.325 ^†^	0.436

Values are presented as mean ± standard deviation or number (%). * *q*-values were adjusted *p*-values for controlling the false discovery rate using the Benjamini–Hochberg method. ^†^ Independent *t*-test; ^‡^ Chi-square test.

**Table 3:** Comparison of clinical outcomes between groups.

	MISTLIF	BETLIF	*p*-Value	*q*-Value *
**Patient number**	90	89		
**Blood transfusion**	13 (14.7%)	0 (0%)	<0.001 ^‡^	<0.001
**Hospital stay (days)**	7.4 ± 2.3	5.7 ± 1.1	<0.001 ^†^	<0.001
**Pre-OP clinical status**				
VAS back pain	5.4 ± 2.8	5.2 ± 3.1	0.651 ^†^	0.772
VAS leg pain	6.2 ± 2.8	6.3 ± 2.5	0.801 ^†^	0.869
ODI	47.5 ± 15.3	46.7 ± 17.0	0.741 ^†^	0.859
JOA score	14.8 ± 5.2	15.6 ± 6.3	0.361 ^†^	0.460
**Post-OP clinical status**				
VAS (back)	1.8 ± 2.1	1.7 ± 2.1	0.750 ^†^	0.850
VAS (leg)	1.9 ± 2.2	1.7 ± 2.0	0.525 ^†^	0.653
ODI	23.5 ± 14.4	12.7 ± 16.1	<0.001 ^†^	<0.001
JOA score	20.6 ± 2.5	26.4 ± 3.2	<0.001 ^†^	<0.001
**MCID attainment rate**				
VAS (back)	66 (73.3%)	75 (84.3%)	0.074 ^‡^	0.140
VAS (leg)	87 (96.7%)	82 (92.1%)	0.187 ^‡^	0.272
ODI	66 (73.3%)	74 (83.1%)	0.112 ^‡^	0.179
JOA score	80 ((88.9%)	87 (97.7%)	0.018 ^‡^	0.042
**Complications**				
Dural tear	1 (1.11%)	1 (1.12%)	0.994 ^‡^	0.994
Pedicle screw malposition	0	2 (2.25%)	0.153 ^‡^	0.230
Epidural hematoma	2 (2.22%)	2 (2.25%)	0.991 ^‡^	1.031
Transient neurological symptom	2 (2.22%)	2 (2.25%)	0.991 ^‡^	1.031
Pedicle screw loosening	3 (3.33%)	0	0.082 ^‡^	0.149
Reoperation	1 (1.11%)	2 (2.25%)	0.554 ^‡^	0.673

Values are presented as mean ± standard deviation or number (%). Pre-OP = pre-operative; Post-OP = post-operative; VAS = visual analogue scale; ODI = Oswestry; Disability Index; JOA = Japanese Orthopedic Association; MCID = minimal clinically important difference. * *q*-values were adjusted *p*-values for controlling the false discovery rate using the Benjamini–Hochberg method. ^†^ Independent *t*-test; ^‡^ Chi-square test.

**Table 4:** Comparison of the functional and radiological results before and after the surgery.

	Pre-OP	Post-OP	*p*-Value	*q*-Value *
**Functional Outcomes**				
**MISTLIF**				
VAS back pain	5.4 ± 2.8	1.8 ± 2.1	<0.001	<0.001
VAS leg pain	6.2 ± 2.8	1.9 ± 2.2	<0.001	<0.001
ODI	47.5 ± 15.3	23.5 ± 14.4	<0.001	<0.001
JOA score	14.8 ± 5.2	20.6 ± 2.5	<0.001	<0.001
**BETLIF**				
VAS back pain	5.2 ± 3.1	1.7 ± 2.1	<0.001	<0.001
VAS leg pain	6.3 ± 2.5	1.7 ± 2.0	<0.001	<0.001
ODI	46.7 ± 17.0	12.7 ± 16.1	<0.001	<0.001
JOA score	15.6 ± 6.3	26.4 ± 3.2	<0.001	<0.001
**Radiological Outcomes**				
**MISTLIF**				
Disc height (mm)	5.9 ± 1.0	9.6 ± 1.0	<0.001	<0.001
Segmental lordosis (°)	7.7 ± 1.8	13.9 ± 4.4	<0.001	<0.001
**BETLIF**				
Disc height (mm)	6.1 ± 1.7	10.5 ± 0.9	<0.001	<0.001
Segmental lordosis (°)	7.3 ± 2.7	12.9 ± 3.7	<0.001	<0.001

Values are presented as mean ± standard deviation. All the analyses use the paired *t*-test. * *q*-values were adjusted *p*-values for controlling the false discovery rate using the Benjamini–Hochberg method.

**Table 5:** Comparison of radiological outcomes between groups.

	MISTLIF	BETLIF	*p*-Value	*q*-Value *
**Patient number**	90	89		
**Fusion segments**	114	114		
**Disc height (mm)**				
Pre-operative	5.9 ± 1.0	6.1 ± 1.7	0.340 ^†^	0.445
Post-operative	9.6 ± 1.0	10.5 ± 0.9	<0.001 ^†^	<0.001
Disc height restoration	3.7 ± 1.5	4.4 ± 1.5	0.004 ^†^	0.005
**Segmental lordosis (°)**				
Pre-operative	7.7 ± 1.8	7.3 ± 2.7	0.246 ^†^	0.339
Post-operative	13.9 ± 4.4	12.9 ± 3.7	0.102 ^†^	0.173
Angle change	6.2 ± 3.7	6.1 ± 2.0	0.822 ^†^	0.937
**Segments with CT scan**	51 (56.7%)	60 (67.4%)	0.140 ^‡^	0.216
**Bridging bone**				
Sagittal plane	45 (88.2%)	58 (96.6%)	0.027 ^‡^	0.053
Coronal plane	42 (82.4%)	56 (93.3%)	0.025 ^‡^	0.053
**Successful fusion**	42 (82.4%)	56 (93.3%)	0.025 ^‡^	0.053
**Bridwell grading**				
Grade I	10 (19.6%)	44 (73.3%)	<0.001 ^‡^	<0.001
Grade II	32 (62.8%)	12 (20.0%)		
Grade III	8 (15.6%)	3 (5.0%)		
Grade IV	1 (2.0%)	1 (1.7%)		
**Subchondral osteolysis**	27 (52.9%)	8 (13.3%)	<0.001 ^‡^	<0.001
**Cage subsidence**				
No subsidence	44 (86.3%)	57 (95.0%)	0.023 ^‡^	0.051 ^‡^
Subsidence	7 (13.7%)	3 (5.0%)		

Values are presented as mean ± standard deviation or number (%). * *q*-values were adjusted *p*-values for controlling the false discovery rate using the Benjamini–Hochberg method. ^†^ Independent *t*-test; ^‡^ Chi-square test.

## Data Availability

The raw data supporting the conclusions of this article will be made available by the authors on request.

## Abbreviations

The following abbreviations are used in this manuscript:

MISTLIF	Minimally invasive transforaminal lumbar interbody fusion
BETLIF	Biportal endoscopic transforaminal lumbar interbody fusion
CT	Computed tomography
MRI	Magnetic resonance image
VAS	Visual analog scale
ODI	Oswestry disability index
JOA	Japanese orthopedic association
UBE	Unilateral biportal endoscopy
MCID	Minimum clinically important difference
FDR	False discovery rate
PEEK	Polyetheretherketone
Ti	Titanium
DBM	Demineralized bone matrix
TCP	Tricalcium phosphate
